# ISO 9001 and JCI preparedness as drivers of quality improvement at a tertiary cardiac care center: a 5-year analysis from Pakistan

**DOI:** 10.1093/intqhc/mzag154

**Published:** 2026-09-22

**Authors:** Huma Shakoor, Waqas Naseem, Syed Shahkar Ahmed Shah, Naveed Shah, Abdul Karim, Tabish Rasool

**Affiliations:** Health Safety & Environment, Peshawar Institute of Cardiology, Peshawar, Pakistan; Department of Health Sciences, University of Debrecen, Debrecen, Hungary; Cardiac Surgery, Peshawar Institute of Cardiology, Peshawar, Pakistan; Quality Assurance Department, Peshawar Institute of Cardiology, Peshawar, Pakistan; Quality Assurance Department, Peshawar Institute of Cardiology, Peshawar, Pakistan; Department of Health Sciences, University of Debrecen, Debrecen, Hungary

**Keywords:** hospital accreditation, ISO 9001 certification, Joint Commission International, quality management system, quality improvement, leadership, cardiac care, low- and middle-income countries

## Abstract

**Background:**

External quality frameworks such as ISO 9001 certification lay the foundation for healthcare quality, patient safety, and operational efficiency, but sustained improvement depends on leadership, a culture of safety, and systems that translate assurance into ongoing learning. Evidence on this transition from low- and middle-income countries, particularly in specialized cardiac care, remains limited.

**Methods:**

A retrospective longitudinal analysis of quality assurance data from January 2020 to December 2025 was conducted at the Peshawar Institute of Cardiology (PIC-MTI), Pakistan. Independent-samples t-tests, one-way ANOVA, and Difference-in-Differences (DiD) modeling were applied, with three regional hospitals of similar bed capacity and patient volume serving as a comparison group.

**Results:**

Following ISO 9001 certification (February 2022), monthly mortality within 48 h declined from 2.8% (SD ± 0.4%) to 1.9% (SD ± 0.3%) (*t* = 3.21, df = 58, *P* = .004). Hospital-acquired infections (HAIs) fell from 3.2% to 2.0% (*t* = 4.15, *P* = .002), reaching 1.3% by 2025, with zero CLABSIs recorded in the final quarter following the mid-period intensification of the infection-control bundle. Mean length of stay decreased from 6.2 to 4.7 days [ANOVA: *F*(2,57) = 8.76, *P* < .001]. Patient satisfaction rose from 78% to above 90%. DiD modeling found a net 30% reduction in HAIs and 20% reduction in length of stay (LOS) associated with the certification process relative to the comparison trajectory.

**Conclusions:**

ISO 9001 certification, undertaken alongside deliberate investment in leadership, a culture of safety, and structured use of data, is associated with sustained improvements in clinical outcomes and operational efficiency at a resource-limited cardiac care center that has not yet pursued formal Joint Commission International (JCI) accreditation but is using JCI’s standards, as one example of an internationally recognized framework, to guide its preparation. The pattern observed here suggests that a learning quality management system, and not any single certificate or accreditation status, is what converts external standards into measurable gains, offering a replicable model for institutions considering how to move from quality assurance toward continual quality improvement.

## Introduction

Accreditation is a voluntary external review process through which healthcare organizations demonstrate a systematic commitment to quality, patient safety, and continuous improvement [1, 2]. Certification standards such as ISO 9001 and the accreditation standards of Joint Commission International (JCI) lay the foundation for this commitment: they provide structured, auditable requirements spanning clinical care, infection control, medication management, and facility safety, and they strengthen risk management and standardize evidence-based practice [3–5]. What neither certification nor accreditation alone can do is guarantee that this foundation is used. As Batalden and Foster describe in their account of a century of healthcare quality work, formal assurance mechanisms have historically preceded, rather than produced, genuine improvement, and the gains that follow depend on whether an organization also builds the leadership commitment, safety culture, and learning systems needed to act on what these external standards make visible [6]. Systematic reviews of the accreditation and certification literature bear this out: these processes are consistently linked to improvements in safety culture, process measures, and efficiency, but effects on clinical outcomes such as mortality and infection rates are more variable and appear to depend heavily on organizational context [7–10].

Accreditation is also not without cost. High implementation expense, sustained staff time, survey preparation burdens, and inconsistent outcome evidence have all been documented [11–13]. Where these programs have produced durable gains, the common thread is not the certificate itself but the governance structures, staff engagement, and data-driven culture that the underlying process requires organizations to build [8, 12].

### Accreditation in Pakistan and the low- and middle-income country context

Most published accreditation evidence originates from high-income countries, with comparatively little data from South Asia [7, 8]. Pakistan carries a growing cardiovascular disease burden that remains the country’s leading cause of mortality and places sustained demand on tertiary cardiac centers operating under resource constraints [14]. Structured quality programs have proven feasible in other resource-constrained health systems, including in Iran and Jordan [4, 12], but longitudinal, quantitatively validated evidence from a cardiac-specific tertiary center in Pakistan has not previously been documented.

### Study objective

This study evaluates the association between ISO 9001 certification, together with the leadership and quality-culture investments that accompanied it, and service quality, patient safety, and operational efficiency at the Peshawar Institute of Cardiology (PIC-MTI) from 2020 to 2025. PIC-MTI has not yet pursued JCI accreditation. Since 2023 it has used JCI standards as a planning framework to guide preparation for a future accreditation survey [15], and this preparatory use of the standards, rather than formal JCI accreditation, is what the analysis below refers to as JCI preparedness. JCI is referenced here only because it is the framework PIC-MTI chose to plan against; JCI is itself accredited by the International Society for Quality in Health Care (ISQua), and the standards of any other ISQua-accredited body could plausibly have served the same planning function. The institution’s ISO 9001 certification predates the 2023 publication of ISO 7101 [16], the newer sector-specific standard for healthcare quality management systems; ISO 7101 is noted in the Discussion as a candidate for future adoption alongside JCI preparation.

## Materials and methods

A retrospective longitudinal design was used to analyze hospital quality assurance data from January 2020 to December 2025. PIC-MTI is a public tertiary cardiac care center in Peshawar, Khyber Pakhtunkhwa, Pakistan, with 337 beds at the end of the study period. The institution was inaugurated in late 2019 and began full clinical operations in January 2020, its inaugural operational year. ISO 9001 certification was achieved in February 2022, following implementation work that began in mid-2020.

Certification was accompanied by parallel investment in governance and culture that is not itself captured in the quantitative indicators below but is relevant to interpreting them. The ISO 9001 process required the institution to formalize executive sponsorship of quality goals, establish a Quality Assurance Department with defined reporting lines to senior leadership, and introduce routine multidisciplinary review of safety data, consistent with evidence that physician–nurse collaboration is a key driver of safety culture in hospital settings [17]. These governance changes preceded and, in the authors’ assessment, enabled the clinical gains described in Results; they are discussed further as a limitation of retrospective attribution below.

Data sources included the Quality Assurance Department’s internal records, patient medical records, ISO 9001 surveillance and audit reports, infection control monitoring logs, and annual institutional performance reports. Performance indicators were benchmarked against published international standards [18]. Patient satisfaction was assessed through quarterly standardized discharge surveys administered to all eligible inpatients, aggregated annually. Infection control adherence was monitored using a structured auditing approach [19, 20], within the organizational framework for infection prevention programs described by Zingg *et al.* [21].

### Statistical analysis

All statistical analysis was performed using SPSS version 25. Descriptive statistics summarized key performance indicators across the study period. Independent-samples t-tests compared monthly performance metrics between the pre-certification period (January 2020 to January 2022, *n* = 25 months) and the post-certification period (March 2022 to December 2024, *n* = 34 months), with pooled data across the full study timeline (*n* = 60 months, df = 58) used for the primary inferential comparisons. A one-way ANOVA assessed the effect of time on average length of stay across three measurement windows (2020, 2022, 2025; between-groups df = 2, within-groups df = 57), with Tukey’s HSD applied for post-hoc pairwise comparisons.

For Difference-in-Differences (DiD) modeling, PIC-MTI’s trajectories were compared against aggregate data from three regional tertiary hospitals in Khyber Pakhtunkhwa that did not undergo ISO 9001 certification during the same period. These comparison figures were drawn from routinely reported, publicly available aggregate indicators rather than from direct collaboration with the hospitals concerned; the hospitals were not informed that their data would be used for this comparison, and for that reason they are not named individually and are reported only as a pooled aggregate trajectory. Comparison hospitals were selected for similarity in bed capacity, patient volume, and clinical specialization; unlike PIC-MTI, they were established institutions that did not undergo a comparable inaugural-to-mature growth trajectory over the study period, which we address as a source of residual confounding in the Limitations. The model controlled for seasonal variation, case mix, and demographic factors. Scenario projections for the anticipated impact of full JCI compliance were derived by applying the observed ISO 9001 DiD effect size to published international benchmarks for JCI-associated gains; these are reported as forward estimates, not confirmed results, since PIC-MTI has not undergone JCI accreditation.

### Ethics

This study involved retrospective analysis of routinely collected, anonymized quality assurance data. No patient identifiers were accessed and no direct patient contact occurred. Under applicable institutional guidelines at PIC-MTI and Pakistan’s national data governance framework, formal ethics committee review is not required for retrospective analyses of anonymized administrative data that do not involve individual-level patient data; this determination was confirmed by institutional quality assurance leadership. Data handling followed national governance standards and the ethical principles of the Declaration of Helsinki as applicable to research using anonymized institutional records.

## Results

### Workforce, infrastructure, and patient volume growth

PIC-MTI grew substantially across all institutional dimensions between 2020 and 2025. Cardiologists increased from 8 in 2020 to 27 at certification and 39 by 2025; cardiac surgeons grew from 4 to 17. Nursing and technical staff expanded in proportion. Bed capacity rose from 255 to 337, catheterization laboratories from 5 to 6, and ICU capacity from 23 to 31 beds. Outpatient visits and cardiac surgical volume rose sharply between the inaugural year (2020) and full operational capacity (2021), reflecting the transition out of the startup phase rather than a single intervention; growth from 2022 onward tracked both expanded capacity and referral volume following ISO 9001 certification.

### Key performance metrics

Table 1 summarizes primary quality indicators across the study period, Figs 1–3 present trend data for mortality, hospital-acquired infection (HAI) rate, and length of stay, and Table 2 sets out the approximate year-on-year sequence of the underlying quality improvement initiatives.

**Figure 1:**
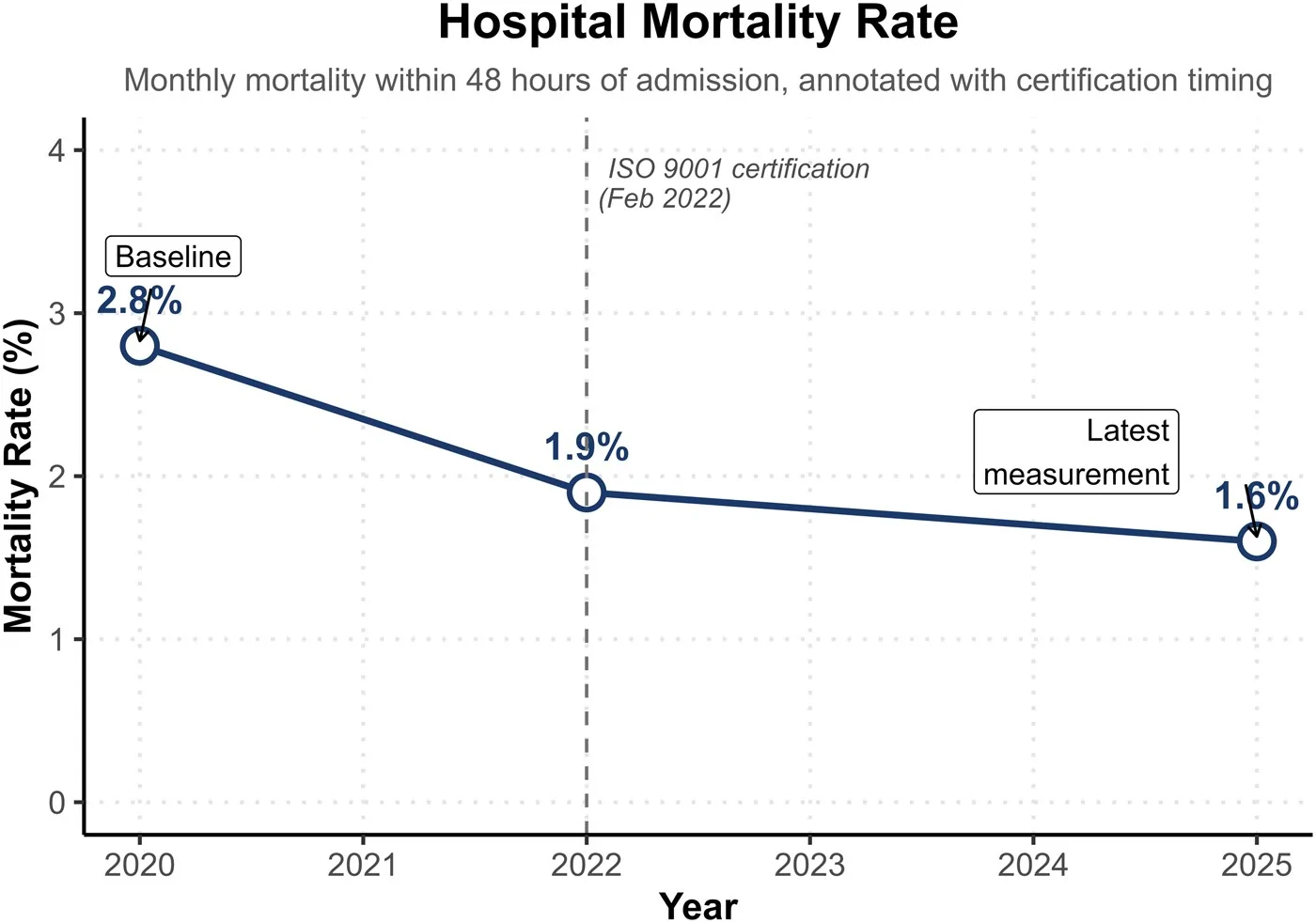
Monthly mortality rate within 48 h of admission at PIC-MTI (2020–5), annotated with ISO 9001 certification date.

**Figure 2:**
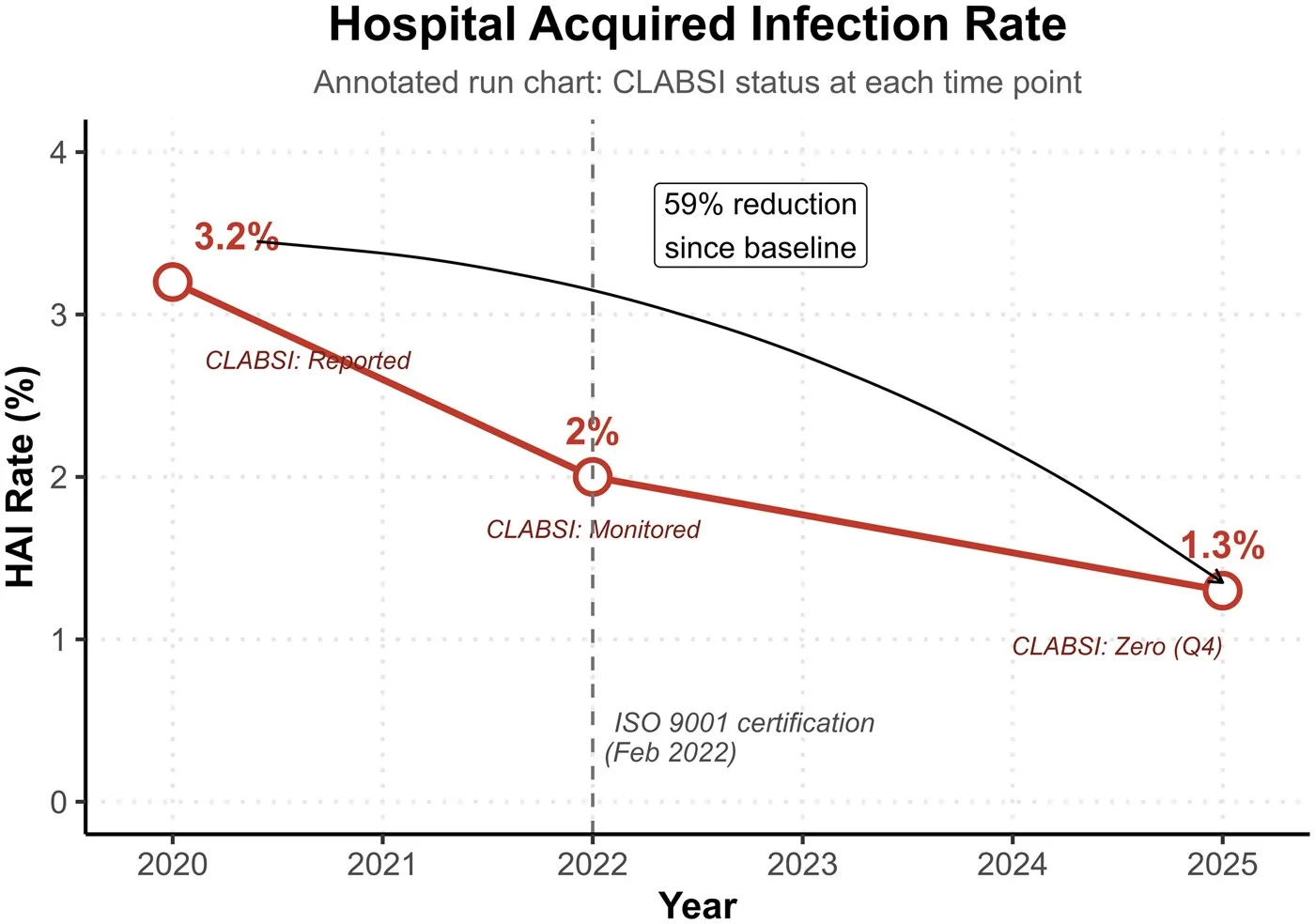
Hospital-acquired infection rate at PIC-MTI (2020–5), annotated run chart showing CLABSI status at each time point.

**Figure 3:**
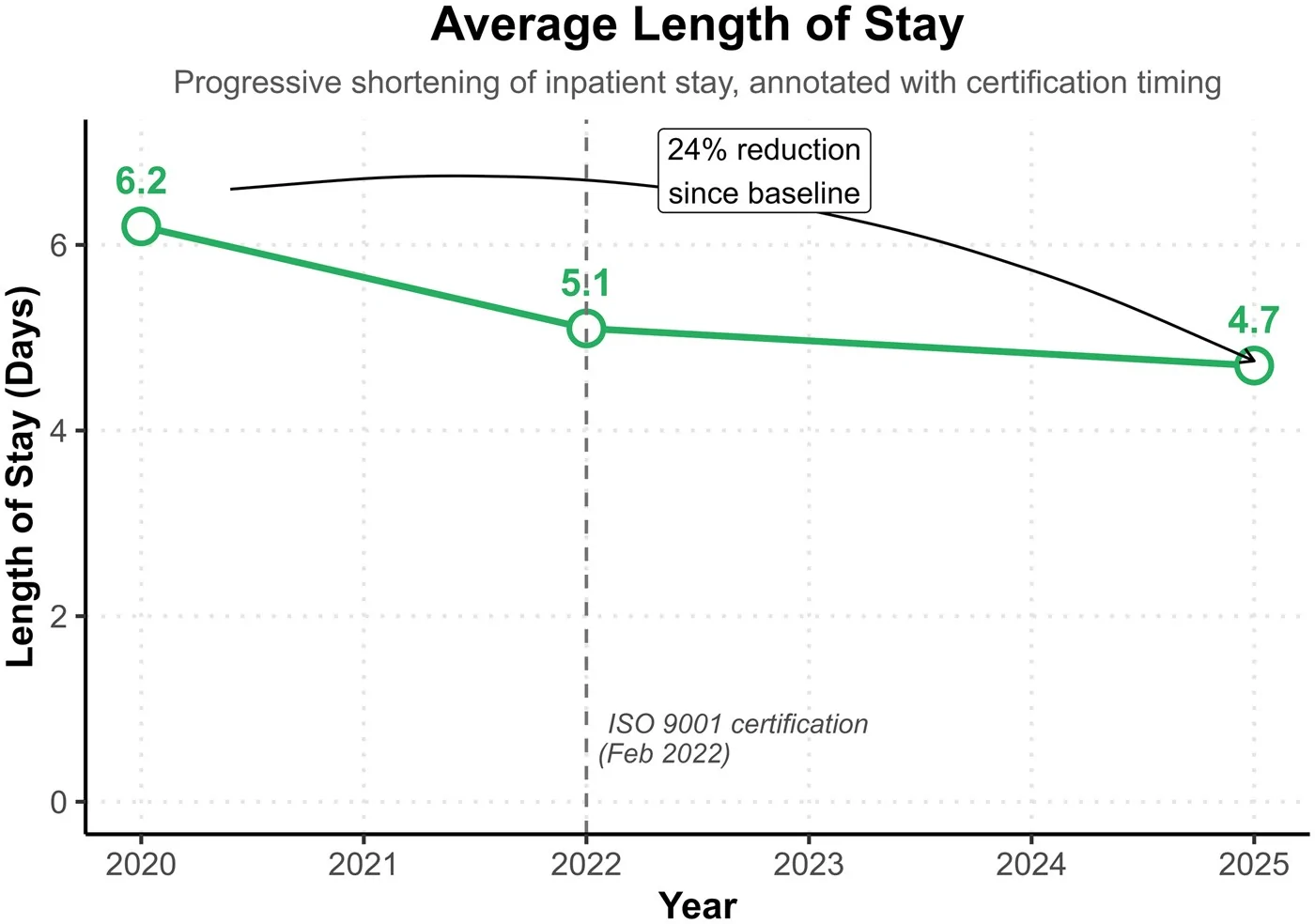
Average length of stay at PIC-MTI (2020–5), annotated with ISO 9001 certification date.

**Table 1:** Key performance metrics at PIC-MTI (2020–5).

Indicator	2020 (pre-ISO)	2021	2022 (post-ISO)	2024 (toward JCI)	2025
Mortality within 48 h (%)	2.8	2.5	1.9	1.7	1.6
Mean LOS (days)	6.2	5.8	5.1	4.9	4.7
Elective surgery wait (days)	32	28	22	18	<15 (85%)
Patient satisfaction (%)	78	82	88	90	>90
HAI rate (%)	3.2	2.8	2.0	1.6	1.3
CLABSI rate	Reported	Declining	Monitored	Low	Zero (Q4)

**Table 2:** Approximate timeline of quality improvement initiatives at PIC-MTI (2020–5)^a^.

Timing	Milestone/initiative
2020	PIC-MTI opens; baseline quality indicators established (CLABSI status: “Reported”).
2020–early 2022	ISO 9001 implementation work under way; initial hand hygiene protocols and infection-control auditing introduced.
February 2022	ISO 9001 certification achieved.
2022–4 (approximate)	Catheter bundle compliance monitoring and structured staff training intensified; CLABSI status moves from “Monitored” toward “Low.”
2023	JCI standards adopted as an internal planning framework for a possible future accreditation survey; ISO 7101:2023 published.
2024	Continued bundle implementation and routine auditing; CLABSI status “Low.”
Q4 2025	Full, routinely audited bundle implementation reached; zero CLABSIs recorded.

a Timing is exact only for ISO 9001 certification, a fixed, recorded date; other milestones are reconstructed from institutional records and the general implementation sequence described in Methods and Results, and should be read as approximate given the retrospective study design (see Strengths and limitations).

### Mortality rate

Monthly mortality within 48 h of admission declined progressively from 2.8% (SD ± 0.4%) in 2020 to 1.6% (95% CI: 1.4%–1.8%) by 2025. The reduction between the pre- and post-certification periods was statistically significant (*t* = 3.21, df = 58, *P* = .004). The sustained downward trend through 2024 and 2025 is consistent with durable rather than transient change (Fig. 1).

### Hospital-acquired infection rate

HAI rates declined from 3.2% in 2020 to 1.3% by 2025, a statistically significant reduction between the pre- and post-certification periods (*t* = 4.15, df = 58, *P* = .002). Zero central line-associated bloodstream infections (CLABSIs) were recorded in the final quarter of 2025. This outcome followed the progressive introduction of hand hygiene protocols, catheter bundle compliance monitoring, staff training, and environmental auditing, all made mandatory and measurable under the ISO 9001 governance framework. Bundle components were introduced incrementally rather than all at once: initial hand hygiene and auditing measures began soon after certification in 2022, with catheter bundle compliance monitoring and structured staff training intensifying around the midpoint of the post-certification period, roughly coinciding with the “Monitored” and “Low” CLABSI stages shown in Table 1, before the bundle reached full, routinely audited implementation ahead of the zero-CLABSI quarter in 2025. The current data allow us to describe this sequence only in general terms; a prospectively collected, annotated statistical process control (SPC) chart, which we did not have available for this retrospective analysis, would be needed to attribute the timing of each specific bundle element to the observed decline with confidence (Fig. 2).

### Length of stay and operational efficiency

Mean length of stay decreased from 6.2 days in 2020 to 4.7 days by 2025. One-way ANOVA showed a significant effect of time [*F*(2,57) = 8.76, *P* < .001], and Tukey’s HSD confirmed 2025 LOS was significantly shorter than both the 2020 baseline (*P* < .001) and the 2022 post-certification estimate (*P* = .014) (Fig. 3). Elective surgical wait times fell from 32 to under 15 days for 85% of cases by 2025, and patient satisfaction rose from 78% to above 90%.

### Statistical summary and DiD analysis

Table 3 presents the full inferential statistics. DiD modeling found a net 30% reduction in HAI rates and a net 20% reduction in LOS associated with ISO 9001 certification relative to the comparison hospital trajectory, after controlling for seasonal variation, case mix, and demographic factors. These effect sizes are larger than those reported in some studies from established health systems; given that the comparison hospitals were mature institutions without PIC-MTI’s concurrent expansion, some of this difference may reflect PIC-MTI’s greater room for improvement as a newly established center rather than certification alone, a point developed further below. Applying the observed ISO 9001 effect magnitude to published JCI outcome benchmarks, scenario projections suggest a further 30% reduction in HAI rates and 20% reduction in LOS with full JCI compliance; these are directional estimates pending formal accreditation and prospective evaluation, not confirmed results.

**Table 3:** Summary of inferential statistics.

Test	Variable	Statistic	df	*P*-value	Interpretation
*T*-test	Mortality rate	*t* = 3.21	58	.004	Significant reduction post-ISO 9001 (2.8%–1.9%)
*T*-test	HAI rate	*t* = 4.15	58	.002	Significant reduction post-ISO 9001 (3.2%–2.0%)
One-way ANOVA	Average LOS	*F* = 8.76	(2,57)	<.001	LOS decreased significantly across all three time periods
DiD (ISO 9001)	HAI rate	Net: –30%	–	<.05	Net reduction associated with certification vs comparison group
DiD (ISO 9001)	Average LOS	Net: –20%	–	<.05	Net reduction associated with certification vs comparison group
Projection (JCI)	HAI rate/LOS	∼30%/∼20%	–	N/A	Scenario estimate based on DiD effect size and JCI benchmarks

## Discussion

### Statement of principal findings

ISO 9001 certification at PIC-MTI was associated with statistically significant, sustained improvement across every monitored domain between 2020 and 2025: mortality, HAI rates, length of stay, elective surgical waiting times, and patient satisfaction.

### Strengths and limitations

This study’s 5-year longitudinal design and inclusion of a DiD comparison group are uncommon in the low- and middle-income country accreditation literature, which relies mostly on cross-sectional or short-term pre-post comparisons. The breadth of indicators monitored, and the clearly defined 2020 inaugural baseline, add to the study’s value.

The central limitation is that this is a retrospective analysis. Before-and-after t-tests and ANOVA can establish that outcomes changed across the study period, but they cannot, on their own, attribute that change to specific dated interventions with the confidence that prospective, annotated SPC data would provide. We have described the general sequence of infection-control bundle implementation in Results, but a fully annotated run chart showing exactly when each intervention was introduced, what its immediate effect was, and where setbacks occurred was not possible with the data available for this retrospective study. This is the most important direction for future work at PIC-MTI. The comparison hospitals, while similar to PIC-MTI in bed capacity and patient volume, were established institutions without PIC-MTI’s concurrent inaugural-to-mature growth trajectory, which limits the precision of causal attribution in the DiD model despite the controls applied. We were also unable to characterize the comparison hospitals’ own leadership, culture, or quality management systems, so the DiD estimates should be read as a secondary check on plausibility rather than as evidence that certification itself, independent of institutional context, produced the difference; the primary evidence in this study is PIC-MTI’s own trajectory before and after certification, which does not depend on the comparability of other hospitals. Patient satisfaction was measured with an internal instrument rather than a validated tool such as Consumer Assessment of Healthcare Providers and Systems (CAHPS), limiting comparability. Some residual confounding from unmeasured variables cannot be excluded.

### Interpretation within the context of the wider literature

These associations are consistent with the broader accreditation and certification literature, which links such processes to improved safety culture and process measures, though effects on clinical outcomes are more variable across settings [7, 8, 22]. The consistent rise in patient satisfaction observed at PIC-MTI is in line with evidence that structured patient-provider communication is a key lever in certification-linked improvement [23].

The pattern of results at PIC-MTI is consistent with the distinction Batalden and Foster draw between quality assurance and quality improvement [6]. ISO 9001 provided the assurance layer: a mandatory, auditable governance structure that made infection control practices, hand hygiene protocols, and staff training measurable and accountable. What appears to have converted that assurance into clinical gain was the parallel investment in leadership sponsorship and a culture of safety described in Materials and methods section, together with the structured patient feedback loop introduced under certification, which is consistent with Hinchcliff and colleagues’ finding that staff engagement and patient feedback integration are central to how accreditation translates into service quality gains [24]. The zero-CLABSI result in the final quarter of 2025 illustrates this pathway: the mechanism was not a single intervention but the cumulative, incrementally introduced embedding of hand hygiene, catheter bundle compliance, staff training, and environmental auditing within a governance framework that ISO 9001 made mandatory [8, 21].

This progression maps closely onto Juran’s quality trilogy of planning, control, and improvement [25]: ISO 9001 supplied the planning and control functions by defining standards and building the audit infrastructure to hold the organization to them, while the sustained leadership push toward zero CLABSI represents the improvement function acting on what those controls made visible.

Viewed through Deming’s system of profound knowledge [26], the same result reflects an appreciation of the hospital as a single system, an understanding of the variation that routine auditing exposed, and leadership prepared to act on that knowledge rather than treat the certificate itself as the end point. Read together, these two frameworks point to the same conclusion as the results reported here: it is the underlying learning quality management system, leadership commitment, a culture of safety, defined standards, improvement initiatives built to meet and exceed them, and structured use of data that produces measurable gains, and any credible external standard can occupy the “standards” position in that system.

The DiD-estimated effect sizes are larger than those reported in some studies from established health systems. This is plausible given PIC-MTI’s greater room for improvement as an institution transitioning from its inaugural phase, but it also means these estimates should be read as associational rather than strictly causal. Before-and-after significance testing of the kind used here, while standard for hospital epidemiology, is not equivalent to the statistical process control methods used in quality improvement work to attribute change to specific, dated interventions [27]. We address this directly in Limitations.

PIC-MTI has not yet pursued formal JCI accreditation. The scenario projections reported here for JCI-associated gains are directional estimates derived from the observed ISO 9001 effect size and published international benchmarks, not confirmed results, and require prospective validation once formal accreditation is pursued. Alongside JCI preparation, the newer ISO 7101:2023 standard, published after PIC-MTI’s ISO 9001 certification and written specifically for management systems in healthcare organizations, may be a relevant complement worth evaluating in future institutional planning [16].

### Implications for policy, practice, and research

The take-home message from PIC-MTI’s experience is that a learning quality management system, not any single certificate or accreditation status, is the foundation of high-quality care. In this study, the system had five recognizable components:

- leadership that sponsored and sustained the work rather than simply signing off on it;
- a culture of quality and safety in which staff at every level treated it as a shared responsibility;
- defined external standards, in this case ISO 9001 and JCI’s framework, against which performance could be planned and audited;
- improvement initiatives built specifically to meet and then exceed those standards, rather than to pass a single survey; and
- structured use of routine data to learn what was working, adjust course, and sustain gains over time.

None of these five components is specific to ISO 9001 or to JCI. The lesson we take from PIC-MTI’s experience for other resource-limited institutions is to invest in this kind of system, and in the leadership and culture that sustain it, from the start, rather than waiting for a particular accreditation survey to prompt it.

From a research perspective, these findings point to clear priorities. Multi-center prospective studies across Khyber Pakhtunkhwa and other provinces would strengthen causal inference. Future work should use validated patient experience instruments such as CAHPS, and should adopt prospective, annotated run or statistical process control charts of the kind described by Provost and Murray, which allow individual interventions to be dated and their effects distinguished from concurrent institutional change [27]. This echoes broader calls in the field for more transparent, innovative approaches to generating accreditation evidence [28]. Prospective evaluation following any future JCI accreditation at PIC-MTI would help separate JCI-specific effects from ongoing institutional maturation.

## Conclusions

ISO 9001 certification, implemented alongside deliberate investment in leadership commitment and a culture of safety, is associated with significant and sustained improvement in mortality, HAI rates, length of stay, and patient satisfaction at PIC-MTI, a resource-limited public cardiac center that was simultaneously undergoing rapid institutional growth. These results are consistent with the view that certification lays a necessary foundation for quality, but the gains described here reflect the fuller set of activities PIC-MTI built on that foundation—leadership, culture, standards, improvement, and data—and not certification alone. Prospective, annotated data collection and multi-center evaluation following any future JCI accreditation are needed to test whether this trajectory can be extended from quality assurance toward the kind of sustained, systems-level improvement that the wider literature associates with mature quality cultures.

## Data Availability

Data are available from the corresponding author upon reasonable request.
